# Sex differences in insulin resistance and associated metabolic and inflammatory markers among adults with and without HIV in Zambia

**DOI:** 10.14814/phy2.71093

**Published:** 2026-09-01

**Authors:** Frederick Sibbenga, David Chisompola, Mumbo Chipuma, Martin Chakulya, Propheria Lwiindi, Masiliso A. Liamba, Benson M. Hamooya, Joreen P. Povia, Situmbeko Liweleya, Sepiso K. Masenga

**Affiliations:** ^1^ HAND Research Group, School of Medicine and Health Sciences Mulungushi University Livingstone Zambia; ^2^ Department of Cardiovascular Science and Metabolic Diseases Livingstone Center for Prevention and Translational Science Livingstone Zambia; ^3^ School of Medicine and Health Sciences University of Lusaka Lusaka Zambia; ^4^ Department of Health Economics Livingstone Center for Prevention and Translational Science Livingstone Zambia; ^5^ Department of Medicine Vanderbilt University Medical Center Nashville Tennessee USA; ^6^ Vanderbilt Center for Immunobiology, Vanderbilt Institute for Infection, Immunology and Inflammation Vanderbilt University Medical Center Nashville Tennessee USA; ^7^ Department of Molecular Physiology and Biophysics Vanderbilt University Medical Center Nashville Tennessee USA; ^8^ Vanderbilt Institute for Global Health Vanderbilt University Medical Center Nashville Tennessee USA

**Keywords:** adiposity, HIV, HOMA‐IR, insulin resistance, renin‐angiotensin system

## Abstract

Insulin resistance (IR) contributes substantially to cardiovascular disease and type 2 diabetes mellitus, with increasing concern among people living with HIV (PLWH). However, sex‐specific factors of IR in sub‐Saharan Africa remain poorly understood. This study evaluated sex‐specific associations between IR and clinical, metabolic, and inflammatory markers among adults with and without HIV in Zambia. A cross‐sectional study was conducted among 233 adults in Zambia. Insulin resistance was assessed using the Homeostatic Model Assessment for Insulin Resistance (HOMA‐IR). Sex‐stratified multivariable linear regression models were used to identify factors associated with IR. Females had higher body mass index (26.9 vs. 23.5 kg/m^2^, *p* < 0.001), waist circumference (88.3 vs. 83.2 cm, *p* = 0.008), fasting insulin, and HOMA‐IR compared with males. In the adjusted overall model, female sex was associated with higher HOMA‐IR (*β* = 0.95, 95% CI: 0.06–1.84; *p* = 0.037), while HIV‐positive status was associated with lower HOMA‐IR (*β* = −1.54, 95% CI: −3.06 to −0.02; *p* = 0.046). Among males, hypertension was associated with higher HOMA‐IR (*β* = 0.61; *p* = 0.019), whereas among females, HIV‐positive status was inversely associated with HOMA‐IR (*β* = −2.31; *p* = 0.049). Insulin resistance highlighted sex‐specific patterns, with greater metabolic risk among females and distinct clinical factors among males. Future studies incorporating lifestyle and hormonal factors are needed to clarify these relationships.

## 1 INTRODUCTION

Insulin resistance (IR) is a central feature of metabolic syndrome and a potent driver of cardiovascular disease (CVD) and type 2 diabetes mellitus (T2DM) (Levy‐Marchal et al., 2010). In the era of modern antiretroviral therapy (ART), people living with HIV (PLWH) are experiencing an increased burden of metabolic disorders, often occurring at younger ages compared to the general population (Moreno‐Lopez et al., 2026). While the transition to integrase strand transfer inhibitor (INSTI)‐based regimens has significantly improved viral suppression and tolerability, it has been associated with disproportionate weight gain and metabolic dysfunction, particularly among women (Alvarez et al., 2024; Nye, 2024).

Biological sex is a critical determinant of metabolic health, influencing adipose tissue distribution, inflammatory responses, and the activity of the renin‐angiotensin system (RAS) (White et al., 2019). Women generally exhibit higher total adiposity but greater peripheral insulin sensitivity compared to men, a protection often attributed to estrogenic effects on glucose metabolism and RAS modulation (Murphy et al., 2018). However, HIV infection and chronic ART exposure may disrupt these sex‐specific metabolic profiles. Emerging evidence suggests that women with HIV experience more pronounced weight gain and altered fat distribution following the initiation of INSTI‐based ART, which may accelerate the development of IR (Alvarez et al., 2024).

The renin‐angiotensin system, particularly its primary effector peptide angiotensin II (Ang II), plays a pivotal role in both vascular and metabolic regulation. Beyond its vasoconstrictive properties, Ang II has been implicated in promoting IR by interfering with insulin signaling pathways and inducing oxidative stress in skeletal muscle and adipose tissue (Srinivasa et al., 2015). In PLWH, RAS activation has been linked to visceral adiposity and systemic inflammation, yet sex‐specific associations between Ang II and IR in this population remain under‐investigated (Shen et al., 2022). Furthermore, the role of pro‐inflammatory cytokines such as interleukin‐17A (IL‐17A) in modulating metabolic risk in HIV is complex, with some studies suggesting a link between Th17‐related inflammation and impaired glucose homeostasis (Bergin et al., 2022).

In Zambia and other sub‐Saharan African settings, evidence describing factors associated with insulin resistance, particularly integrating HIV status, inflammatory markers, RAAS activation, renal function, and sex‐specific factors, remains limited. Understanding these relationships is important for improving cardiometabolic risk assessment and informing targeted prevention strategies among populations experiencing a dual burden of infectious and non‐communicable diseases.

Therefore, this study aimed to evaluate factors associated with insulin resistance, measured using HOMA‐IR, among adults, and to determine whether these associations differ between males and females. This study was designed as an exploratory, hypothesis‐generating investigation to examine sex‐stratified associations between insulin resistance and clinical, metabolic, and inflammatory markers among adults with and without HIV in Zambia.

## 2 METHODOLOGY

### 2.1 Study design and population

This study was conducted at the medical outpatient clinic of Livingstone University Teaching Hospital between 25 August 2023 and 30 April 2024. We employed a cross‐sectional study design to conduct an exploratory investigation of the determinants of insulin resistance among adults with and without HIV.

The study was conducted in accordance with the principles of the Declaration of Helsinki (2013) and relevant Zambian national ethical guidelines. Ethical approval was obtained from the Mulungushi University School of Medicine and Health Sciences Research Ethics Committee on 09 July 2023 (Ref. SMHS‐MU3‐2023‐005), with additional administrative clearance granted by Livingstone University Teaching Hospital. All participants provided written informed consent prior to enrollment.

The study population consisted of adult participants aged 18 years and above who attended the medical clinic for routine care or follow‐up and provided written informed consent. Both people living with HIV (PLWH) and HIV‐negative individuals were included to enable comparative analysis. Eligible participants with HIV infection were required to have been on antiretroviral therapy for at least 6 months and to have achieved viral suppression, defined as a viral load of less than 50 copies/mL.

Participants were recruited consecutively during clinic visits over the study period. Individuals with conditions that could significantly influence metabolic or inflammatory markers, including pregnancy, active malignancy, acute infections, severe renal or hepatic disease, and opportunistic infections, were excluded to minimize potential confounding.

### 2.2 Rationale for inclusion of HIV‐negative participants

HIV‐negative individuals were included to serve as a comparator group, providing context for understanding whether associations observed in PLWH are specific to HIV infection or reflect broader population‐level determinants of insulin resistance. Given the high HIV prevalence in Zambia and the shared environmental and socioeconomic factors, inclusion of HIV‐negative participants enhances the interpretability of findings by allowing comparison of HOMA‐IR distributions and predictor associations across HIV status (Mulenga et al., 2024).

### 2.3 Sample size

The sample size for this study was determined pragmatically based on the number of eligible participants available during the study period at Livingstone University Teaching Hospital. A total of 233 participants were recruited. Although a formal a priori sample size calculation was not performed, the sample size of 233 participants was considered adequate for exploratory sex‐stratified multivariable analyses based on the number of covariates included in the models and feasibility within the study period (Moradi et al., 2023; Msollo et al., 2019).

### 2.4 Blood sampling and laboratory testing

Participants were approached during routine clinic visits, and those who agreed to participate and provided written informed consent were scheduled for a follow‐up appointment on a separate date, during which they were instructed to fast for at least 8–12 h prior to sample collection.

Venous blood samples were then collected using standard aseptic techniques. Approximately 5–10 mL of blood was drawn into appropriate collection tubes, including:

- Fluoride oxalate tubes (gray top) for glucose measurement, containing sodium fluoride as a glycolysis inhibitor and potassium oxalate as an anticoagulant to preserve glucose concentrations by preventing glycolysis.
- EDTA (lavender/purple top) for complete blood count and hematological parameters, containing ethylenediaminetetraacetic acid as an anticoagulant to chelate calcium and preserve cellular morphology.
- Serum separator tubes (SST, red/gold top) for biochemical and immunological analyses, containing a gel barrier that separates serum from cellular components upon centrifugation, with no anticoagulant to allow for serum collection.

Immediately after collection, tubes were gently inverted 6–8 times to mix the contents with any additives; tubes were not shaken to avoid hemolysis. All samples were labeled with unique study identifiers, date, and time of collection. Cotton or gauze was applied to the venipuncture site with pressure for 1–2 min to achieve hemostasis, and a bandage was applied.

### 2.5 Sample processing and storage

Samples were processed within 2 h of collection; plasma and serum were separated by centrifugation at 3000 rpm for 10 min at room temperature using a benchtop centrifuge.

Following centrifugation, the supernatant (plasma from EDTA tubes, serum from SST tubes) was carefully aliquoted using sterile pipettes into 2 mL cryovials labeled with study identifiers. For EDTA tubes, plasma was collected from the top layer, taking care not to disturb the buffy coat layer containing white blood cells and platelets. For SST tubes, serum was collected from the top layer above the gel barrier. All aliquots were immediately stored at −80°C in a monitored freezer until analysis to preserve the stability of analytes, including proteins, hormones, and inflammatory markers.

Fasting glucose and lipid profiles were measured using automated clinical chemistry analyzers, while fasting insulin concentrations were quantified using immunoassay‐based methods. Insulin resistance was estimated using the Homeostatic Model Assessment for Insulin Resistance (HOMA‐IR). Inflammatory markers, including high‐sensitivity C‐reactive protein (hs‐CRP) and interleukin‐17A (IL‐17A), as well as angiotensin II levels, were measured using commercially available enzyme‐linked immunosorbent assay (ELISA) kits Elabscience (Elabscience Biotechnology Inc., Houston, USA) in accordance with the manufacturers' instructions. The detection ranges for IL‐17A (E‐UNEL‐H0002) were 31.25–2000 pg/mL, while hs‐CRP (E‐EL‐H5134) was 15.63–1000 pg/mL, and angiotensin II (E‐EL‐H0326) was 31.25–2000 pg/mL. The intra‐assay coefficients of variation (CVs) were <8%, and inter‐assay CVs were <10%, consistent with acceptable assay performance thresholds reported in biomarker studies (typically <10%). Furthermore, each sample was assayed in duplicate (two technical replicates per sample) for all ELISA assays to minimize pipetting error and ensure reproducibility.

All assays were performed with appropriate internal quality controls, and laboratory procedures adhered to standard operating protocols to ensure accuracy and reproducibility.

### 2.6 Outcome variable: Insulin resistance (HOMA‐IR)

The primary outcome of this study was insulin resistance, assessed using the Homeostatic Model Assessment for Insulin Resistance (HOMA‐IR). HOMA‐IR was calculated from fasting plasma glucose and fasting insulin concentrations using the standard formula:

$$\text{HOMA}-\mathrm{IR}=\frac{\text{Fasting insulin}\;\left(\mathrm{\mu U}/\mathrm{mL}\right)\times \text{Fasting glucose}\;\left(\text{mmol}/L\right)}{22.5}$$

Participants with a HOMA‐IR ≥1.9 were classified as insulin resistant (Simon et al., 2020). HOMA‐IR was analyzed as a continuous variable in both univariable and multivariable linear regression models.

HOMA‐IR was selected as the outcome variable because it is a widely validated surrogate marker of insulin resistance with strong correlation to the hyperinsulinemic‐euglycemic clamp, the gold standard method for assessing insulin sensitivity (Chauhan et al., 2026). It is particularly suitable for epidemiological studies because it requires only fasting glucose and insulin measurements, making it feasible in resource‐limited settings. HOMA‐IR has also been validated across diverse populations, including people living with HIV (Hulgan, 2018). Although it primarily reflects hepatic insulin resistance and is influenced by variability in insulin assays, it remains a practical and reliable tool for identifying insulin resistance patterns in population‐based research.

### 2.7 Data collection

Data were collected using standardized procedures by trained research personnel at Livingstone University Teaching Hospital. Sociodemographic and clinical information was obtained through structured interviews and review of medical records using a pre‐designed electronic data capture tool (REDCap). Variables collected included age, sex, marital status, employment status, smoking history, and medical history such as HIV status, hypertension, and prior tuberculosis. For participants living with HIV, additional information on antiretroviral therapy (ART) regimen and duration of treatment was extracted from clinical records.

A calibrated stadiometer was employed to carefully measure height (expressed in meters) and weight (quantified in kilograms), and body mass index (BMI) was calculated as the ratio of weight to height squared (kg/m^2^). Waist circumference was measured at the midpoint located between the inferior border of the ribs and the iliac crest.

Clinical measurements, including blood pressure, were obtained using validated automated devices after participants had rested for at least 5 min in a seated position. All measurements were performed in duplicate, and the average values were used for analysis. Data quality was ensured through routine checks for completeness and consistency, and all data were securely stored with restricted access to maintain participant confidentiality.

### 2.8 Statistical analysis

Data were exported from REDCap and analyzed using Stata version 16. Continuous variables were assessed for distributional normality using histograms, Q Q plots, and the Shapiro Wilk test. Normally distributed continuous variables were summarized as means and standard deviations, whereas skewed variables were summarized as medians and interquartile ranges. Categorical variables were summarized as frequencies and percentages.

Baseline characteristics were compared by sex using the Students *t*‐test for normally distributed continuous variables, the Mann Whitney *U* test for skewed continuous variables, and the chi square test or Fishers exact test for categorical variables, as appropriate.

HOMA IR was used as the primary outcome and treated as a continuous variable. Because HOMA‐IR and selected biomarkers demonstrated right‐skewed distributions, logarithmic transformation was performed prior to regression analyses to improve model normality and homoscedasticity assumptions. Univariable linear regression was first used to examine associations between HOMA IR and candidate covariates. Multivariable linear regression models were then fitted for overall population and then stratified by sex as males and females to evaluate sex specific factors associated with insulin resistance. Covariates were selected a priori on the basis of biological relevance and existing literature, including Sex, HIV status, hypertension, triglycerides, estimated glomerular filtration rate, angiotensin II and IL 17A.

Multicollinearity was assessed using variance inflation factors (VIF). Model assumptions were evaluated by inspection of residual plots, tests for heteroscedasticity, and assessment of influential observations. Where heteroscedasticity was detected, robust standard errors were used. Regression results are reported as beta coefficients with 95 percent confidence intervals and *p*‐values. A two‐sided *p*‐value less than 0.05 was considered statistically significant.

## 3 RESULTS

### 3.1 Participants characteristics

A total of 233 participants were recruited. 79 (33.9%) were males while 154 (66.1%) were females. The age distribution appears comparable between males and females (median ~48 years, *p* = 748), suggesting minimal age‐related confounding across sex groups. However, a pronounced socioeconomic disparity is evident, with unemployment disproportionately higher among females compared to males (70.4% vs. 60.8%, *p* = 0.139). Marital status shows a striking divergence: males are predominantly married (70.9%), whereas females are largely unmarried (69.1%) (*p* < 0.001). With respect to HIV status, a higher proportion of females were living with HIV compared to males (63.6% vs. 55.7%, *p* = 240) (Table 1). Notably, the overwhelming majority of participants are on INSTI‐based regimens in both males and females. Clinically, females showed a less favorable adiposity profile, with higher BMI (26.9 vs. 23.5 kg/m^2^, *p* < 0.001) and waist circumference (88.3 vs. 83.2 cm, *p* = 0.008), indicating greater central obesity (Table 1). This is accompanied by higher fasting insulin levels and HOMA‐IR, suggesting a stronger burden of insulin resistance among females (*p* < 0.05). Biochemically, most lipid parameters appear broadly similar; however, subtle differences emerge in glucose metabolism, with females exhibiting slightly elevated fasting glucose. Inflammatory markers (hs‐CRP and IL‐17A) trend higher in females, hinting at a heightened inflammatory milieu, although variability is substantial (*p* > 0.05). Interestingly, males exhibit markedly higher ALT levels, suggesting potential sex‐specific hepatic or metabolic stress, while electrolyte and renal markers remain broadly comparable across groups. The prevalence of insulin resistance was higher among females compared to males (17.5 vs. 12.7, *p* = 0.335).

**TABLE 1:** Baseline demographic, clinical, and biochemical characteristics of the study participants.

Variable	Male 79 (33.9)	Female 154 (66.1)	*p*‐value
	*n* (%) or mean (SD)	*n* (%) or mean (SD)	
Age, years, IQR	48.4 (13.8)	47.6 (13.9)	0.748
Employed status			
Employed	31 (39.2)	45 (29.6)	0.139
Unemployed	48 (60.8)	107 (70.4)	
Marital status			
Married	56 (70.9)	47 (30.9)	**<0.001**
Not married	23 (29.1)	105 (69.1)	
HIV Status			
Positive	44 (55.7)	98 (63.6)	0.240
Negative	35 (44.3)	56 (36.4)	
ART regimen			
NNRTI + NRTI	0 (0)	4 (4.8)	0.227
INSTI (TLD or TafED)	39 (100)	77 (92.8)	
PIs	0 (0)	2 (2.4)	
History of tuberculosis			
Yes	9 (14.1)	9 (12.9)	0.824
No	55 (85.9)	108 (87.1)	
Hypertension status			
Hypertensive	19 (24.1)	44 (28.6)	0.462
Non‐hypertensive	60 (75.9)	110 (71.4)	
Smoking			
Yes	7 (9.1)	5 (3.4)	0.114
No	70 (90.9)	142 (96.6)	
Insulin resistance			
Sensitive	69 (87.3)	127 (82.5)	0.335
Resistant	10 (12.7)	27 (17.5)	
BMI (kg/m^2^)	23.5 (4.6)	26.9 (5.8)	**<0.001**
Waist circumference (cm)	83.2 (12.9)	88.3 (14.8)	**0.008**
HDL cholesterol (mmol/L)	1.2 (0.5)	1.2 (0.4)	0.379
Triglycerides (mmol/L)	1.0 (0.7)	0.9 (0.6)	0.821
Fasting glucose (mmol/L)	4.8 (0.8)	5.1 (1.2)	0.082
Fasting insulin (μU/mL)	4.2 (4.8)	6.3 (9.6)	**0.020**
HOMA‐IR	0.9 (1.1)	1.6 (3.1)	**0.024**
Alanine aminotransferase (U/L)	33.2 (71.8)	17.3 (13.0)	**<0.001**
Plasma potassium (mmol/L)	4.1 (0.5)	6.7 (0.4)	0.270
Plasma chloride (mmol/l)	102.1 (4.1)	103.5 (5.6)	**0.024**
Plasma sodium (mmol/l)	145.3 (2.4)	144.7 (2.7)	0.341
Creatinine, (μmol/l)	95.7 (80.2)	85.1 (90.7)	**0.009**
eGFR (mL/min/1.73 m^2^)	118.0 (54.6)	108.8 (70.6)	**0.013**
Hs‐CRP log (pg/mL)	4.3 (1.3)	4.4 (1.4)	0.776
IL‐17A (pg/mL)	252.2 (587.9)	491.4 (1168.1)	0.278

*Note*: Socioeconomic variables such as employment status may act as indirect factors associated with metabolic risk through behavioral and healthcare access pathways. INSTI: Integrase Strand Transfer Inhibitor‐based antiretroviral therapy; reflects current WHO‐recommended first‐line regimens. HOMA‐IR calculated as a surrogate index of insulin resistance using fasting glucose and insulin. Values presented as mean (SD). ART regimen percentages were calculated among participants living with HIV receiving ART. Bold values represent statistical significance.

### 3.2 Simple linear regression analysis for factors associated with HOMA‐IR in males

**FIGURE 1:**
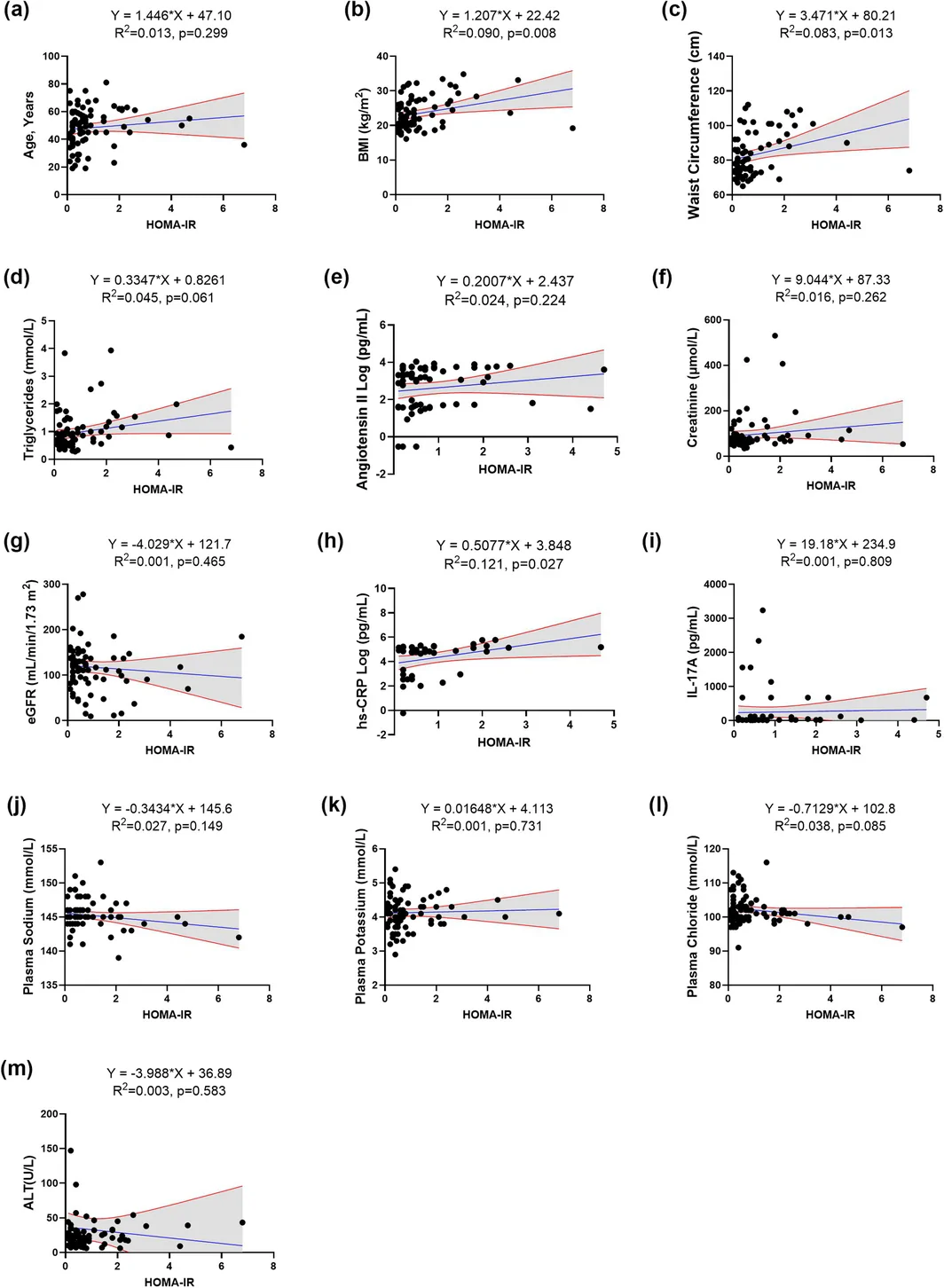
Simple linear regression analysis for factors associated with HOMA‐IR in males.

Scatter plots (A–M) illustrate the relationship between the Homeostatic Model Assessment for Insulin Resistance and selected demographic, anthropometric, biochemical, inflammatory, and renal variables in the overall study population. Panels include: (A) age, (B) body mass index (BMI), (C) waist circumference, (D) angiotensin II, (E) triglycerides, (F) creatinine, (G) estimated glomerular filtration rate (eGFR), (H) plasma sodium, (I) plasma potassium, (J) plasma chloride, (K) interleukin‐17A (IL‐17A), (L) log‐transformed high‐sensitivity C‐reactive protein (hs‐CRP), and (M) alanine aminotransferase (ALT) (Figure 1). Linear regression lines (blue) with 95% confidence intervals (red shading) are shown for each association, with corresponding regression equations, coefficients of determination (*R* ^2^), and *p*‐values displayed within each panel. Overall, BMI, waist circumference, and hs‐CRP were positively associated with HOMA‐IR (*p* < 0.05). However, age, triglycerides, angiotensin II, creatinine, eGFR, plasma sodium, plasma potassium, plasma chloride, IL‐17A, and ALT were not associated with HOMA‐IR (Figure 1).

### 3.3 Simple linear regression analysis for factors associated with HOMA‐IR in females

**FIGURE 2:**
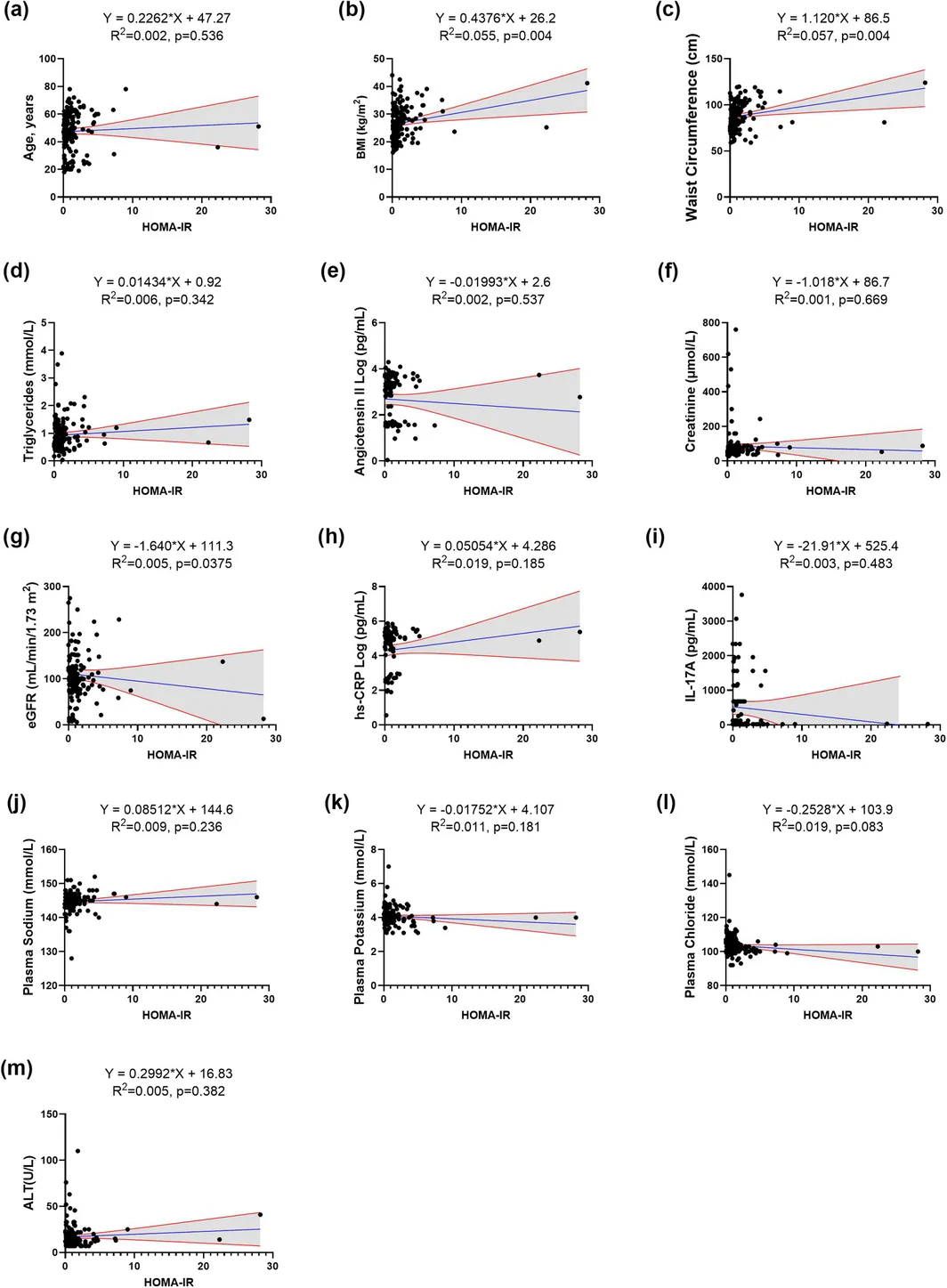
Simple linear regression analysis for factors associated with HOMA‐IR in females.

Scatter plots (A–M) depict the associations between Homeostatic Model Assessment for Insulin Resistance and selected variables among people living with HIV (PLHIV). Variables include: (A) age, (B) BMI, (C) waist circumference, (D) triglycerides, (E) angiotensin II, (F) creatinine, (G) eGFR, (H) log‐transformed hs‐CRP, (I) IL‐17A, (J) plasma sodium, (K) plasma potassium, (L) plasma chloride, and (M) ALT (Figure 2). Overall BMI and waist circumference were associated with HOMA‐IR (*p* < 0.05). However, age, triglycerides, angiotensin II, creatinine, eGFR, plasma sodium, plasma potassium, plasma chloride, hs‐CRP, IL‐17A, and ALT were not associated with HOMA‐IR (Figure 2).

### 3.4 Factors associated with HOMA‐IR

In the overall unadjusted linear regression analyses of factors associated with HOMA‐IR, female sex (*β* = 0.63, 95% CI: −0.80 to 1.34, *p* = 0.080), HIV‐positive status (*β* = −0.59, 95% CI: −1.28 to 0.10, *p* = 0.091), and hypertension (*β* = 0.73, 95% CI: −0.03 to 1.48, *p* = 0.060) revealed borderline associations with HOMA‐IR, while triglycerides, eGFR, log‐transformed angiotensin II, and log‐transformed IL‐17A were not significantly associated with the outcome (Table 2).

**TABLE 2:** Simple and Multiple Linear regression of factors associated with HOMA‐IR.

Parameter	Unadjusted		Adjusted	
	*β* (95% CI)	*p*‐value	*β* (95% CI)	*p*‐value
Sex (female)	0.63 (−0.80, 1.34)	0.080	0.95 (0.06, 1.84)	**0.037**
HIV status (positive)	−0.59 (−1.28, 0.10)	0.091	−1.54 (−3.06, −0.02)	**0.046**
Hypertension status (hypertensive)	0.73 (−0.03, 1.48)	0.060	0.79 (−0.19, 1.76)	0.114
Triglycerides (mmol/L)	0.39 (−0.19, 0.97)	0.183	0.65 (−0.13, 1.41)	0.100
eGFR (mL/min/1.73 m^2^)	−0.003 (−0.008, 0.002)	0.235	−0.004 (−0.011, 0.002)	0.170
Angiotensin II log (pg/mL)	−0.05 (−0.35, 0.26)	0.760	0.44 (−0.13, 1.01)	0.133
IL‐17A log (pg/mL)	−0.20 (−0.69, 0.29)	0.427	−0.32 (−0.95, 0.31)	0.317

*Note*: *β* = regression coefficient representing change in HOMA‐IR per unit increase in a factor. CI = confidence interval; values in parentheses represent 95% confidence intervals. Bold values indicate statistical significance (*p* < 0.05). Log transformation applied to skewed variables (Angiotensin II and IL‐17A). Adjusted models include variables significant at univariate level (*p* < 0.05) and covariates selected a priori based on biological plausibility, prior literature, and known relevance to insulin resistance. These included age, sex, HIV status, hypertension status, triglycerides, estimated glomerular filtration rate (eGFR), angiotensin II, and IL‐17A.

In the adjusted model, female sex was independently associated with higher HOMA‐IR compared with males (*β* = 0.95, 95% CI: 0.06 to 1.84, *p* = 0.037). Conversely, HIV‐positive status was independently associated with lower HOMA‐IR (*β* = −1.54, 95% CI: −3.06 to −0.02, *p* = 0.046). Hypertension (*β* = 0.79, 95% CI: −0.19 to 1.76, *p* = 0.114), triglycerides (*β* = 0.65, 95% CI: −0.13 to 1.41, *p* = 0.100), eGFR (*β* = −0.004, 95% CI: −0.011 to 0.002, *p* = 0.170), log‐transformed angiotensin II (*β* = 0.44, 95% CI: −0.13 to 1.01, *p* = 0.133), and log‐transformed IL‐17A (*β* = −0.32, 95% CI: −0.95 to 0.31, *p* = 0.317) were not independently associated with HOMA‐IR (Table 2). Multicollinearity was assessed using VIF; the mean VIF was 1.76, indicating no significant multicollinearity among covariates.

### 3.5 Factors associated with HOMA‐IR males

In unadjusted linear regression analyses of factors associated with HOMA‐IR, none of the variables reached conventional statistical significance, although hypertension status and triglyceride levels showed borderline associations. Hypertensive males had higher HOMA‐IR than normotensive males (*β* = 0.57, 95% CI: −0.01 to 1.16, *p* = 0.053), while higher triglyceride concentrations were associated with increased HOMA‐IR (*β* = 0.35, 95% CI: −0.02 to 0.73, *p* = 0.061). HIV status (*β* = −0.02, 95% CI: −0.53 to 0.49, *p* = 0.939), eGFR (*β* = −0.001, 95% CI: −0.006 to 0.003, *p* = 0.465), log‐transformed angiotensin II (*β* = 0.12, 95% CI: −0.08 to 0.32, *p* = 0.224), and log‐transformed IL‐17A (*β* = 0.26, 95% CI: −0.08 to 0.61, *p* = 0.136) were not significantly associated with HOMA‐IR (Table 3).

**TABLE 3:** Simple and multiple linear regression of factors associated with HOMA‐IR males.

Parameter	Unadjusted		Adjusted	
	*β* (95% CI)	*p*‐value	*β* (95% CI)	*p*‐value
HIV status (positive)	−0.02 (−0.53, 0.49)	0.939	−0.47 (−1.27, 0.32)	0.239
Hypertension status (hypertensive)	0.57 (−0.01, 1.16)	0.053	0.61 (0.11, 1.11)	**0.019**
Triglycerides (mmol/L)	0.35 (−0.02, 0.73)	0.061	0.35 (−0.03, 0.74)	0.074
eGFR (mL/min/1.73 m^2^)	−0.001 (−0.006, 0.003)	0.465	−0.01 (−0.010, −0.001)	**0.029**
Angiotensin II log (pg/mL)	0.12 (−0.08, 0.32)	0.224	0.16 (−0.17, 0.48)	0.335
IL‐17A log (pg/mL)	0.26 (−0.08, 0.61)	0.136	0.14 (−0.26, 0.54)	0.485

*Note*: *β* = regression coefficient representing change in HOMA‐IR per unit increase in a factor. CI = confidence interval; values in parentheses represent 95% confidence intervals. Bold values indicate statistical significance (*p* < 0.05). Log transformation applied to skewed variables (Angiotensin II and IL‐17A). Adjusted models include variables significant at univariate level (*p* < 0.05) and covariates selected a priori based on biological plausibility, prior literature, and known relevance to insulin resistance. These included sex, HIV status, hypertension status, triglycerides, estimated glomerular filtration rate (eGFR), angiotensin II, and IL‐17A.

In the adjusted model, hypertensive males had significantly higher HOMA‐IR than normotensive males (*β* = 0.61, 95% CI: 0.11 to 1.11, *p* = 0.019). In contrast, higher eGFR was independently associated with lower HOMA‐IR (*β* = −0.01, 95% CI: −0.010 to −0.001, *p* = 0.029), suggesting that better kidney function was associated with reduced insulin resistance. HIV status remained unrelated to HOMA‐IR after adjustment (*β* = −0.47, 95% CI: −1.27 to 0.32, *p* = 0.239). Similarly, triglycerides (*β* = 0.35, 95% CI: −0.03 to 0.74, *p* = 0.074), log‐transformed angiotensin II (*β* = 0.16, 95% CI: −0.17 to 0.48, *p* = 0.335), and log‐transformed IL‐17A (*β* = 0.14, 95% CI: −0.26 to 0.54, *p* = 0.485) were not independently associated with HOMA‐IR (Table 3). Multicollinearity was assessed using VIF; the mean VIF was 1.77, indicating no significant multicollinearity among covariates.

### 3.6 Factors associated with HOMA‐IR females

In the unadjusted analysis, HIV‐positive status showed a borderline inverse association with HOMA‐IR (*β* = −0.98, 95% CI: −2.00 to 0.03, *p* = 0.057). Hypertension (*β* = 0.75, 95% CI: −0.33 to 1.83, *p* = 0.175), triglyceride levels (*β* = 0.44, 95% CI: −0.48 to 1.36, *p* = 0.342), eGFR (*β* = −0.003, 95% CI: −0.01 to 0.003, *p* = 0.376), log‐transformed angiotensin II (*β* = −0.12, 95% CI: −0.54 to 0.30, *p* = 0.574), and log‐transformed IL‐17A (*β* = −0.42, 95% CI: −1.10 to 0.25, *p* = 0.220) were not significantly associated with HOMA‐IR (Table 4).

**TABLE 4:** Simple and multiple linear regression of factors associated with HOMA‐IR females.

Parameter	Unadjusted		Adjusted	
	*β* (95% CI)	*p*‐value	*β* (95% CI)	*p*‐value
HIV status (positive)	−0.98 (−2.0, 0.03)	0.057	−2.31 (−4.30, −0.01)	**0.049**
Hypertension status (hypertensive)	0.75 (−0.33, 1.83)	0.175	0.77 (−0.55, 2.10)	0.251
Triglycerides (mmol/L)	0.44 (−0.48, 1.36)	0.342	0.71 (−0.44, 1.87)	0.225
eGFR (mL/min/1.73 m^2^)	−0.003 (−0.01, 0.003)	0.376	−0.004 (−0.01, 0.03)	0.291
Angiotensin II log (pg/mL)	−0.12 (−0.54, 0.30)	0.574	0.64 (−1.97, 1.48)	0.132
IL‐17A log (pg/mL)	−0.42 (−1.10, 0.25)	0.220	−0.45 (−1.33, 0.42)	0.311

*Note*: *β* = regression coefficient representing change in HOMA‐IR per unit increase in a factor. CI = confidence interval; values in parentheses represent 95% confidence intervals. Bold values indicate statistical significance (*p* < 0.05). Log transformation applied to skewed variables (hs‐CRP, Angiotensin II, IL‐17A). Adjusted models include variables significant at univariate level (*p* < 0.05) and covariates selected a priori based on biological plausibility, prior literature, and known relevance to insulin resistance. These included sex, HIV status, hypertension status, triglycerides, estimated glomerular filtration rate (eGFR), angiotensin II, and IL‐17A.

After adjustment for covariates, HIV‐positive status was independently associated with lower HOMA‐IR among females (*β* = −2.31, 95% CI: −4.30 to −0.01, *p* = 0.049), indicating that women living with HIV had significantly lower insulin resistance compared with HIV‐negative women. None of the remaining variables were independently associated with HOMA‐IR. Hypertension (*β* = 0.77, 95% CI: −0.55 to 2.10, *p* = 0.251), triglycerides (*β* = 0.71, 95% CI: −0.44 to 1.87, *p* = 0.225), eGFR (*β* = −0.004, 95% CI: −0.01 to 0.03, *p* = 0.291), log‐transformed angiotensin II (*β* = 0.64, 95% CI: −1.97 to 1.48, *p* = 0.132), and log‐transformed IL‐17A (*β* = −0.45, 95% CI: −1.33 to 0.42, *p* = 0.311) remained non‐significant in the adjusted model (Table 4). Multicollinearity was assessed using VIF; the mean VIF was 1.95, indicating no significant multicollinearity among covariates.

## 4 DISCUSSION

This study provides a preliminary, exploratory investigation of factors associated with insulin resistance, measured by HOMA‐IR, among adults, with additional analyses stratified by sex. In the overall population, female sex was independently associated with higher HOMA‐IR, whereas HIV‐positive status was associated with lower HOMA‐IR after adjusting for potential confounders. Sex‐stratified analyses revealed distinct patterns, with hypertension and reduced eGFR independently associated with higher HOMA‐IR among males, while HIV‐positive status was inversely associated with HOMA‐IR among females. These findings suggest that factors associated with insulin resistance may differ according to sex and HIV status, highlighting the importance of considering biological and clinical differences when evaluating metabolic risk.

The observation that females had higher HOMA‐IR compared with males in the adjusted combined model is consistent with evidence that sex‐related biological factors influence glucose metabolism and insulin sensitivity (Anaya‐Ambriz et al., 2025; Wani et al., 2025). Women and men differ in body fat distribution, hormonal regulation, inflammatory responses, and adipose tissue function, all of which may affect insulin resistance (Gado et al., 2024). Although females could typically show greater subcutaneous fat deposition than men, hormonal changes across the life course, particularly during the menopausal transition, together with increasing adiposity and metabolic inflammation, may contribute to higher insulin resistance in some populations (Gusev et al., 2026). The finding of higher HOMA‐IR among females emphasizes the need for sex‐specific approaches when assessing metabolic complications, particularly in populations affected by HIV and chronic inflammation.

In the combined model, HIV‐positive status was independently associated with lower HOMA‐IR compared with HIV‐negative participants. This inverse association was particularly evident among females, where HIV‐positive women had significantly lower HOMA‐IR after adjustment. These findings differ from reports describing increased insulin resistance among people living with HIV due to chronic immune activation, antiretroviral therapy exposure, mitochondrial dysfunction, and altered lipid metabolism (Hulgan, 2018). However, previous studies have also reported variable effects of HIV on glucose metabolism, influenced by ART regimens, duration of infection, nutritional status, body composition, and population characteristics (Hamooya et al., 2026; Willig & Overton, 2016). The lower HOMA‐IR observed among HIV‐positive participants in this study may therefore reflect differences in ART exposure, lifestyle factors, body composition, or other unmeasured factors affecting metabolic regulation.

Among male participants, hypertension was independently associated with higher HOMA‐IR after adjustment. This finding supports the well‐established bidirectional relationship between hypertension and insulin resistance (Castro et al., 2023). Insulin resistance promotes endothelial dysfunction, increased sympathetic nervous system activity, oxidative stress, and impaired vascular regulation, which may contribute to elevated blood pressure (Martinez‐Dominguez et al., 2025; Młynarska et al., 2025). Conversely, hypertension‐related vascular abnormalities may worsen metabolic dysfunction. Interestingly, this association was not statistically significant among females or in the combined model, suggesting that sex‐specific factors, differences in sample size, hormonal influences, or metabolic profiles may influence the relationship between hypertension and insulin resistance.

Reduced eGFR was independently associated with higher HOMA‐IR among males, indicating a potential link between impaired renal function and metabolic abnormalities. Kidney dysfunction is increasingly recognized as both a consequence and contributor to insulin resistance through factors involving chronic inflammation, oxidative stress, altered insulin clearance, and disturbances in glucose metabolism (Gao et al., 2025; Parvathareddy et al., 2023). The absence of a significant association in females and in the overall model may suggest that the relationship between renal function and insulin sensitivity is modified by sex or influenced by other metabolic factors. Further studies are required to clarify whether kidney‐related metabolic alterations differ between men and women.

Neither angiotensin II nor IL‐17A was independently associated with HOMA‐IR in the adjusted analyses. This finding contrasts with experimental evidence suggesting that activation of the renin–angiotensin system and inflammatory pathways contribute to insulin resistance through oxidative stress, impaired insulin signaling, and immune‐mediated metabolic dysfunction (Liu, 2007; Underwood & Adler, 2013). The absence of significant associations may reflect the complexity of these pathways, where circulating biomarker concentrations may not fully capture tissue‐level activity. Additionally, the cross‐sectional nature of the analysis limits inference regarding temporal relationships between inflammation, RAAS activation, and insulin resistance.

The sex‐stratified analyses revealed different patterns of association; however, these differences should be interpreted cautiously because formal interaction testing was not performed. The presence of statistical significance in one sex but not the other does not necessarily confirm effect modification. Future analyses incorporating sex‐by‐factor interaction terms are needed to determine whether HIV status, hypertension, kidney function, or inflammatory markers have significantly different effects on insulin resistance between males and females.

The relatively low means HOMA‐IR values observed in this cohort warrant consideration. Similar findings have been reported in other Sub‐Saharan African populations and may reflect population‐specific differences in body composition, dietary patterns, physical activity, genetic background, and the lower prevalence of obesity‐related insulin resistance compared with Western populations (Kitilya et al., 2022). Furthermore, commonly used HOMA‐IR thresholds derived from European and North American populations (typically ≥2.5–3.0) may not be directly applicable to African populations due to differences in metabolic profiles and underlying risk factors (Wang et al., 2025).

Despite the relatively low absolute HOMA‐IR values, the observed significant associations with clinical factors, particularly sex, hypertension, and HIV status, highlight the biological and clinical relevance of HOMA‐IR as a marker of insulin resistance.

### 4.1 Strengths and limitations

This study contributes to a limited but growing body of literature examining sex‐specific metabolic risk in African populations, particularly in the context of HIV. The inclusion of both immunological (IL‐17A), hormonal (Angiotensin II), and traditional metabolic markers provides a comprehensive framework for understanding insulin resistance beyond conventional risk factors.

However, several limitations must be acknowledged. The cross‐sectional design precludes causal inference, and the observed associations should be interpreted as correlational. A key limitation of this study is the relatively small sample size for the sex‐stratified analyses, particularly among males (*n* = 79), which may have reduced statistical power and increased the risk of model overfitting. Consequently, the adjusted estimates should be interpreted with caution, as smaller samples may produce less stable regression coefficients and wider confidence intervals. Nevertheless, these analyses were exploratory and provide preliminary evidence on sex‐specific factors associated with HOMA‐IR that should be confirmed in larger, adequately powered studies. Additionally, the use of HOMA‐IR, while practical, is an indirect measure of insulin resistance and may not fully capture tissue‐specific insulin sensitivity.

Another major limitation is the absence of data on physical activity, dietary intake, and menopausal status. These are well‐established factors associated with insulin resistance, and their omission introduces substantial risk of residual confounding. Physical activity and dietary patterns may differ substantially by sex in our Zambian setting, potentially explaining the observed sex‐stratified patterns. Menopausal status is particularly critical given the median female age of 48 years, as the menopausal transition profoundly affects glucose metabolism and inflammation. Without these data, we cannot determine whether the observed associations reflect biological sex differences, lifestyle factors, hormonal status, or interactions among these variables. We therefore interpret our findings as exploratory and hypothesis‐generating, emphasizing the need for future studies that include comprehensive assessment of these confounders.

## 5 CONCLUSION

This study revealed factors associated with insulin resistance differ according to sex among adults. In the overall population, female sex was associated with higher HOMA‐IR, while HIV‐positive status was associated with lower HOMA‐IR after adjustment. Sex‐stratified analyses identified hypertension and renal function as important correlates of insulin resistance among males, whereas HIV‐positive status showed an inverse association with HOMA‐IR among females. These findings suggest that metabolic risk pathways may differ between men and women and highlight the importance of incorporating sex‐specific considerations into the assessment and management of insulin resistance, particularly in populations affected by HIV.

However, given the absence of important factors such as physical activity, dietary intake, and menopausal status, the observed sex‐specific associations should be interpreted cautiously. Future studies integrating comprehensive lifestyle, hormonal, inflammatory, and treatment‐related assessments are required to clarify the factors underlying these relationships and to determine whether the observed differences represent true biological sex effects or reflect differences in environmental and physiological factors.

## AUTHOR CONTRIBUTIONS

**Frederick Sibbenga:** Conceptualization; data curation; validation; visualization. **David Chisompola:** Conceptualization; data curation; formal analysis; methodology; validation; visualization. **Mumbo Chipuma:** Data curation; validation; visualization. **Martin Chakulya:** Validation; visualization. **Propheria Lwiindi:** Validation; visualization. **Masiliso A. Liamba:** Formal analysis; validation; visualization. **Benson M. Hamooya:** Formal analysis; validation; visualization. **Joreen P. Povia:** Validation; visualization. **Situmbeko Liweleya:** Supervision; validation; visualization. **Sepiso K. Masenga:** Funding acquisition; project administration; supervision; validation; visualization.

## FUNDING INFORMATION

This work was supported by the Fogarty International Center and National Institute of Diabetes and Digestive and Kidney Diseases of the National Institutes of Health grants R01HL144941, R21TW012635 (S.K.M.), the International Federation of Clinical Chemistry and Laboratory Medicine's Task Force on Outcome Studies in Laboratory Medicine (TF‐OSLM) (S.K.M.) and the American Heart Association Award Number 24IVPHA1297559 (S.K.M.). The content is solely the responsibility of the authors and does not represent the official views of the National Institutes of Health, IFCC, and the American Heart Association. The funders had no role in the study design, data collection and analysis, decision to publish, or preparation of the manuscript.

## CONFLICT OF INTEREST STATEMENT

The authors have no conflicts of interest to declare.

## ETHICS STATEMENT

Ethical approval for the study was obtained from the Mulungushi University School of Medicine and Health Sciences Research Ethics Committee on 09 July 2023 (Ref. SMHS‐MU3‐2023‐005), with additional administrative clearance granted by Livingstone University Teaching Hospital. The study was conducted in accordance with the principles of the Declaration of Helsinki (2013) and relevant national ethical guidelines. All data were anonymized at the point of collection, and no information capable of identifying individual participants was recorded or retained.

## CONSENT

Written informed consent was obtained from all participants prior to enrollment. Informed consent was obtained from the participants for publication.

## Data Availability

The datasets used and/or analyzed during the current study are available from the corresponding author on reasonable request.
